# Epilepsy genetics in Africa: a systematic review

**DOI:** 10.3389/fgene.2026.1964157

**Published:** 2026-10-28

**Authors:** Salia Bamba, Abdoulaye Yalcouyé, Lassana Cissé, Stephen Kamuli, Modibo K. Goita, Salimata Diarra, Guida Landouré, Saquib A. Lakhani

**Affiliations:** 1 Faculté de Médecine et D’Odontostomatologie, Université des Sciences, des Techniques et des Technologies de Bamako, Bamako, Mali; 2 Department of Pediatrics, Cedars Sinai Guerin Children’s, Los Angeles, CA, United States; 3 McKusick-Nathans Institute and Department of Genetic Medicine, School of Medicine, Johns Hopkins University, Baltimore, MD, United States; 4 Service de Médecine Générale, Hôpital Nianankoro Fomba, Ségou, Mali; 5 Service de Neurologie, Centre Hospitalier Universitaire Du Point G, Bamako, Mali

**Keywords:** Africa, DNA, epilepsy, exome sequencing, genetics, genome sequencing, variants

## Abstract

**Background:**

While our understanding of genetic epilepsies is growing, data from Africa remain scant. In this study, we reviewed the profile of epilepsy genetics in Africa.

**Methods:**

We searched for studies in PubMed, Scopus, Google Scholar, and African Journals Online up to December 2025 using a combination of terms related to “epilepsy”, “genetics”, and “Africa”, and extracted data including phenotype and genetic diagnostics.

**Results:**

Seventy-eight studies from 12 countries met our criteria, encompassing 1,858 individuals (1,339 unaffected, 519 affected) from 384 families. The vast majority of families were reported from three countries, Egypt (144/384, 37.5%), South Africa (76/384, 19.8%), and Morocco (60/384, 15.6%). Generalized epilepsy was the most common presentation (30.5%), followed by focal (18.5%), and unspecified (10.4%). Exome sequencing was used in 71.8% of studies and functional validation was performed in 17.9% of studies (n = 14/78), including cell-based models (12.8%, n = 10/78) and animal models (5.1%, n = 4/78). A total of 165 unique diagnostic genes were reported. Genetic heterogeneity was high, with only 12 genes shared across three or more countries. Of the 384 families, 76.3% (n = 293/384) had pathogenic or likely pathogenic variants, with the remaining 23.7% (n = 91/384) having VUSs in genes consistent with the seizure phenotype. Six founder variants accounted for 10.7% of the families, 62.0% of variants were novel at the time of reporting, and 67.8% were classified as pathogenic or likely pathogenic. Consanguinity was 45.6% with autosomal recessive the most common inheritance pattern (49.5%).

**Conclusion:**

This study highlights substantial clinical and genetic heterogeneity of epilepsy in Africa with population-specific founder variants. Notably, only a limited number of African countries (12/54) have published on molecular diagnostics for epilepsy. The data suggest the potential for significant discoveries yet to be made but also point to ongoing disparities in the availability of genomic testing.

## Introduction

Epilepsy is a complex brain disorder and one of the most common neurological disorders globally, affecting approximately 50 million individuals, with nearly 80% of the cases in developing countries (Klein et al., 2024). In Africa alone, an estimated 25 million people are affected, making it a significant public health challenge (Biset et al., 2024). Notably, epilepsy is not just a medical issue but a source of profound social, cultural, psychological, and economic consequences. Furthermore, around 60% of individuals with epilepsy lack access to adequate anti-epileptic treatment, which exacerbates the disease burden (Duta et al., 2025; GBD Epilepsy Collaborators, 2025).

Epilepsy is also clinically heterogeneous. The current ‘International League Against Epilepsy’ (ILAE) classification categorized seizures as focal, generalized seizures, unknown, or unclassified seizures (Karakis, 2025). Overall, classification has significantly evolved over time underscoring its complexity. Similarly, regarding epilepsy type, it is now recognized as focal epilepsy, generalized epilepsy, combined generalized and focal epilepsy, or unknown epilepsy type (Specchio et al., 2022). It integrates clinical, EEG, and imaging data to guide diagnosis and management. However, it is worth noting that this classification does not encompass neonatal seizures, as a separate classification has been proposed for that (Pressler et al., 2021).

While acquired causes including stroke, head trauma, brain infections, and metabolic disorders are predominant, genetic variants are increasingly recognized as key determinants of various epilepsy syndromes (Shlobin et al., 2021). Current data suggest that although the cause of epilepsy remains unknown in 40%–50% of cases, approximately 30% may be attributed to genetic variants (Berkovic et al., 2006; Begley et al., 2022; Ademuwagun et al., 2025; Bamba et al., 2025; Bamba et al., 2024; Cisse et al., 2024; Kerkeni et al., 2025; Lyahyai et al., 2018; Amllal et al., 2024). Recent curated catalogs list more than 1,506 epilepsy-associated genes including primary and syndromic epilepsy (Zhang et al., 2024).

Emerging genomic sequencing efforts have begun uncovering novel variants and candidate genes in some parts of Africa (Yeetong et al., 2024; Landoure et al., 2014; Kassahun Bekele et al., 2023; Samia et al., 2022; Quansah and McGregor, 2018). To date, there is no comprehensive study that accurately captures the genetic landscape of epilepsy in Africa, which is needed to provide improved epilepsy care and develop targeted therapies (Kassahun Bekele et al., 2023; Landouré et al., 2025).

To address this gap, we systematically reviewed studies reporting genetically determined epilepsy in Africa.

## Methodology

### Study design

The protocol of this systematic review was not prospectively registered in PROSPERO. We conducted the review according to the PRISMA guidelines for transparent reporting of systematic reviews and meta-analyses to ensure rigor, transparency, and reproducibility (Page et al., 2021).

### Research question

What is the current genetic landscape of epilepsy in Africa?

### Objectives

Identify and synthesize evidence on the clinical features and associated genetic characteristics of epilepsy in Africa.

### Search strategy

We performed a comprehensive literature search in the following databases: PubMed (https://pubmed.ncbi.nlm.nih.gov/), Scopus (https://www.scopus.com/search/form.uri?display=basic&zone=header&origin=#basic), African Journals Online (AJOL) (https://www.ajol.info/index.php/ajol), and Google Scholar (https://scholar.google.com/), as previously described (Yalcouyé et al., 2022). We used the following combination of Medical Subject Heading (MeSH) terms and keywords: (“epilepsy” OR “genetic epilepsy” OR “epilepsy genetics” OR “hereditary epilepsy” OR “inherited epilepsy” OR “monogenic epilepsy” OR “familial epilepsy” OR “seizure disorder” OR “epileptic syndrome” OR “epileptic encephalopathy”) AND (“Africa” OR “Sub-Saharan Africa” OR “North Africa”). The structured search strategy was designed to identify any published articles that report clinical and genetic data of epilepsy in African countries. Only articles published in English and French were considered.

### Selection criteria

We included original research articles, case reports, cohort studies, and genetic studies published from database inception until 31 December 2025, that reported data on the clinical and genetic features of epilepsy in Africa. In case of duplicate studies, we selected the most recent and/or most informative studies. We excluded qualitative, susceptibility and association studies, commentary and opinion, and review articles. Also, studies with unavailable full text or missing key data were excluded from this review. In the case of articles reporting on both African and non-African patients, we extracted the African patients’ data as done previously (Yalcouyé et al., 2022).

### Selection of studies

All titles, abstracts and full-text articles were screened independently by two reviewers (SB and AY). Disagreements were resolved through discussion and consensus. We employed an automated screening workflow using coding scripts for search, removal of duplication, and preliminary keyword-based triage (scripts are provided in Supplementary Material). In addition, data were manually screened to check their accuracy. All records were downloaded and imported into EndNote. Full-text eligibility was assessed using predefined inclusion and exclusion criteria, and reasons for exclusion were documented. In addition, we did manual data harmonization to appropriately categorize selected clinical characteristics, including seizure classification using the ILAE guidelines and electroencephalogram interpretation (Specchio et al., 2022), before proceeding to summary statistics.

### Data extraction

A standardized data extraction sheet was used to collect information, including first author’s name (used for study identification), year of publication, country of patient origin, sample size, demographic data, and detailed clinical and genetic characteristics. All the variables collected are provided in Supplementary Material. Seizure classification was recorded as ‘Not reported’ when the source publication did not specify seizure semiology; this category reflected a data-reporting gap in the literature and not a diagnostic category. Age at seizure onset and diagnosis was categorized into three groups: Neonates & Infants (birth to < 2 years), Childhood (2–17 years), and Adulthood (≥ 18 years). Additionally, a genetic heterogeneity score was calculated by dividing the total number of families carrying a pathogenic or likely pathogenic variant in a particular gene by the total number of unique genes identified in the overall cohort. For each variant, we extracted the reported zygosity and inheritance pattern (autosomal dominant, autosomal recessive, X-linked, *de novo*, or unknown) as stated in the original publication. Variant classifications per ACMG criteria were retained in the dataset as reported in the source publication. Variants of Uncertain Significance (VUS) that were reported as a candidate variant for the proband’s epilepsy were treated as diagnostic. Incidental findings were not reported.

### Risk of bias and quality assessment

To assess the methodological quality of the included studies, we applied the Joanna Briggs Institute (JBI) Critical Appraisal Checklist for Case Reports (8 items) to case reports (n = 66) and the JBI Critical Appraisal Checklist for Case Series (10 items) to case series (n = 11). Each item was scored as Yes (1 point), Unclear (0.5 point), No (0 points), or Not Applicable (excluded from the denominator), and studies were classified as high quality (≥ 75%), moderate quality (50%–74%), or low quality (< 50%) (Supplementary Table S1).

## Results

### Study selection, quality and population details

We initially identified 1,667 records, and then 34 duplicates were removed. A total of 1,633 records were eligible for title and abstract screening, following which 141 full-text articles were assessed for eligibility. We then removed review articles and studies where epilepsy was not the primary or secondary phenotype. Ultimately, 78 studies met the inclusion criteria and were included in the final analysis. As per PRISMA guidelines, the study selection process is provided in Figure 1A.

**FIGURE 1:**
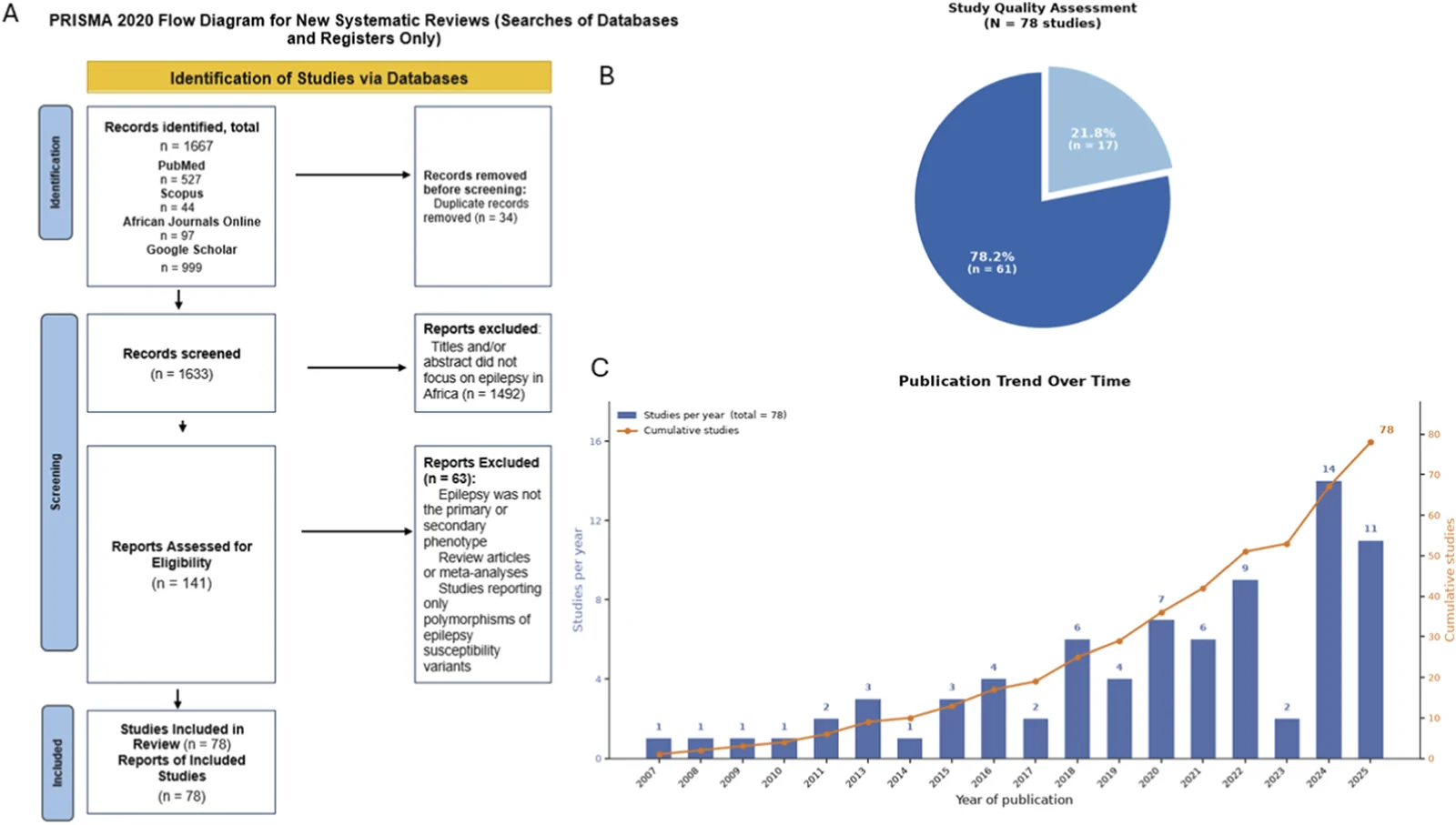
Study selection, quality assessment, and publication trends. **(A)** PRISMA 2020 flow diagram illustrating the systematic literature search and study selection process. A total of 1,667 records were identified from four databases (PubMed, Scopus, African Journals Online, and Google Scholar). **(B)** Pie chart depicting the quality distribution of the 78 included studies, assessed using a standardized quality appraisal tool. **(C)** Bar and line graph showing the publication trend of included studies over time (2007–2025). Blue bars represent the number of studies published per year (total = 78), and the orange line represents cumulative study count, demonstrating a marked increase in research output from year 2019 onward.

Overall, the methodological quality of included studies was acceptable. The mean JBI score was 79% (range: 56%–95%). Most studies, 78.2% (n = 61 of 78) were rated high quality and 21.8% (n = 17 of 78) were of moderate quality (Figure 1B). In case reports, the outcome after treatment was only described in 42% (n = 28 of 66) and side effects or unexpected events were not reported in any study (Supplementary Table S1). Figure 1C shows the dynamics of epilepsy genetics research in Africa over time.

The included studies were published between 2007 and 2025 and comprised 1,858 individuals, including 519 patients with epilepsy of genetic origin from 384 families, along with 1,339 unaffected relatives. There were 270 males and 248 females (sex ratio of 1.1:1), with one study not specifying the sex of the individual (Supplementary Table S2). Most studies, 89.7% (n = 70/78), were case series or reports, while cohort studies represented 10.3% (n = 8/78). All studies were hospital-based. Studies were reported from 22.2% of African countries (n = 12/54), distributed as follows: Egypt, 32.1% (n = 25/78), Morocco, 20.5% (n = 16/78), Tunisia, 16.7% (n = 13/78), Mali, 7.7% (n = 6/78), South Africa, 6.4% (n = 5/78), Sudan, 5.1% (n = 4/78), Algeria, 3.8% (n = 3/78), Libya, 2.6% (n = 2/78), Mauritania, 1.3% (n = 1/78), Rwanda, 1.3% (n = 1/78), Nigeria, 1.3% (n = 1/78), and Tanzania, 1.3% (n = 1/78). The largest number of families were from Egypt, 37.5% (n = 144/384), South Africa, 19.8% (n = 76/384), Morocco, 15.6% (n = 60/384), Tunisia, 12.2% (n = 47/384), and Mali, 8.1% (n = 31/384). The characteristics of the families and the specific studies that described these data are provided in Supplementary Tables S1, S2.

### Epilepsy phenotype characteristics

The summary statistics of the phenotypes are provided in Table 1, and all specific details about the phenotype per family are reported in Supplementary Table S2. A total of 384 families with molecular evidence were included. Consanguinity was present in 45.6% (n = 175/384) of families, absent in 33.6% (n = 129/384), and not reported in the remaining 20.8% (n = 80/384). Consanguinity was mostly reported from North Africa, including Morocco, Egypt, Tunisia, and Algeria. A positive family history of epilepsy was documented in 21.1% (n = 81/384) of cases with only one affected individual in 78.9% (n = 303/384). The age at onset was neonates and infants, 52.3% (n = 201/384), childhood, 28.1% (n = 108/384), adulthood, 1.3% (n = 5/384), and was not reported in 18.2% (n = 70/384).

**TABLE 1:** Clinical characteristics of African families with epilepsy included in this study (384 families, 78 studies, 12 African countries).

Country	Families (n)	Individuals Affected / Unaffected (n)	Clinical characteristics				Neurodevelopmental profile		Treatment			Main comorbidities
			Age at seizure onset	Age at diagnosis	Seizure types (ILAE)	Epilepsy syndrome	Developmental delay	Drug resistance	Anti-seizure medications (ASM)	EEG Findings	Neuroimaging	
Algeria	3	14 / 8	Childhood: 2 (67%) NR: 1 (33%) Range: 2-9 yr (med. 6)	Childhood: 2 (67%) Adulthood: 1 (33%) Range: 2-19 yr (med. 6)	Generalized: GTC (2) Focal: (1)	Progressive Myoclonic Epilepsy (2) Unspecified (1)	Yes: 3 No: 0 NR: 0	Yes: 2 No: 0 NR: 1	Clonazepam (1) Phenobarbital (1) Levetiracetam (1) Valproate (1)	Normal: 0 Abnormal: 2 NR: 1	Normal: 1 Abnormal: 0 NR: 2	Ataxia (2) Progressive cognitive decline (1)
Egypt	144	555 / 199	Neonates & Infants: 91 (63%) Childhood: 40 (28%) Adulthood: 2 (1%) NR: 11 (8%) Range: Birth 36 yr (med. 1)	Neonates & Infants: 37 (26%) Childhood: 95 (66%) Adulthood: 11 (8%) NR: 1 (1%) Range: Birth 36 yr (med. 3)	Generalized: Spasms/IS (64), GTC (30), Myoclonic (28), Tonic (25), Atonic (12) Focal: (42)	DEE (68) Generalized epilepsy (37) Focal epilepsy (23) West syndrome (8)	Yes: 144 No: 0 NR: 0	Yes: 31 No: 3 NR: 110	Valproate (20) Levetiracetam (11) Phenobarbital (8) Lamotrigine (8) Clonazepam (7) Unspecified (101)	Normal: 29 Abnormal: 94 NR: 21	Normal: 45 Abnormal: 92 NR: 7	ID/Cognitive impairment (144) Microcephaly (45) Hypotonia (38) Ophthalmological issues (22)
Libya	2	2 / 2	Childhood: 1 (50%) NR: 1 (50%) Range: 3 yr	Childhood: 1 (50%) NR: 1 (50%) Range: 2 yr	Generalized: GTC (1), Spasms/IS (1)	Generalized epilepsy (1) Unspecified (1)	Yes: 1 No: 1 NR: 0	Yes: 1 No: 0 NR: 1	Hydrocortisone (1) Vigabatrin (1) Unspecified (1)	Normal: 1 Abnormal: 1 NR: 0	Normal: 0 Abnormal: 2 NR: 0	Global developmental delay (1) Hypotonia (1)
Mali	31	130 / 54	Neonates & Infants: 10 (32%) Childhood: 14 (45%) Adulthood: 1 (3%) NR: 6 (19%) Range: Birth 37 yr (med. 3)	Neonates & Infants: 5 (16%) Childhood: 16 (52%) Adulthood: 9 (29%) NR: 1 (3%) Range: Birth 46 yr (med. 8)	Generalized: GTC (22), Myoclonic (8), Spasms (2), Clonic (2) Focal: (4)	Progressive Myoclonic Epilepsy (7) Generalized epilepsy (16) DEE (4) Focal epilepsy (4)	Yes: 31 No: 0 NR: 0	Yes: 9 No: 0 NR: 22	Sodium valproate (9) Clonazepam (8) Levetiracetam (4) Carbamazepine (3) Piracetam (2) Unspecified (21)	Normal: 1 Abnormal: 7 NR: 23	Normal: 4 Abnormal: 3 NR: 24	Developmental delay (31) Ataxia (5) Progressive cognitive decline (4) Myoclonic jerks (4)
Mauritania	1	2 / 2	Childhood: 1 (100%) Range: 3 yr	Childhood: 1 (100%) Range: 5 yr	Focal: (1) Generalized: Myoclonic (1), Tonic (1)	Focal epilepsy (1)	Yes: 1 No: 0 NR: 0	Yes: 1 No: 0 NR: 0	Valproic acid (1) Clonazepam (1) Topiramate (1)	Normal: 0 Abnormal: 1 NR: 0	Normal: 1 Abnormal: 0 NR: 0	Infantile epileptic encephalopathy (1) Developmental delay (1)
Morocco	60	205 / 70	Neonates & Infants: 26 (43%) Childhood: 13 (22%) NR: 21 (35%) Range: Birth 7 yr (med. 2)	Neonates & Infants: 19 (32%) Childhood: 40 (67%) NR: 1 (2%) Range: 1-17 yr (med. 4)	Generalized: GTC (8), Tonic (10), Spasms (9), Myoclonic (7), Absence (7), Atonic (5) Focal: (15) Mixed/other: (23)	Generalized epilepsy (22) DEE (15) Focal epilepsy (10) Dravet syndrome (6) PME (5)	Yes: 60 No: 0 NR: 0	Yes: 15 No: 1 NR: 44	Sodium valproate (9) Levetiracetam (5) Lamotrigine (2) Clonazepam (2) Unspecified (51)	Normal: 12 Abnormal: 25 NR: 23	Normal: 21 Abnormal: 18 NR: 21	Developmental delay (60) Intellectual disability (18) Dysmorphic features (8) Hypotonia (7)
Nigeria	5	15 / 5	Neonates & Infants: 2 (40%) Childhood: 3 (60%) Range: Birth-16 yr (med. 4)	Childhood: 1 (20%) Adulthood: 4 (80%) Range: 4-56 yr (med. 39)	Generalized: Spasms/IS (3), Myoclonic (1), GTC (2) Focal: (2)	Generalized epilepsy (4) DEE (3) Dravet syndrome (1)	Yes: 5 No: 0 NR: 0	Yes: 5 No: 0 NR: 0	Sodium valproate (5) Levetiracetam (4) Pyridoxine (2) Prednisolone (2) Clobazam (2)	Normal: 0 Abnormal: 3 NR: 2	Normal: 0 Abnormal: 1 NR: 4	Global developmental delay (5) Speech/language delay (4) Motor delay (3) Visual impairments (2)
Rwanda	1	1 / 1	Childhood: 1 (100%) Range: 2 yr	Childhood: 1 (100%) Range: 2 yr	Unspecified	Unspecified	Yes: 1 No: 0 NR: 0	Yes: 0 No: 0 NR: 1	NR	Normal: 0 Abnormal: 0 NR: 1	Normal: 0 Abnormal: 0 NR: 1	Developmental delay (1) Facial dysmorphism (1) Hypotonia (1)
South Africa	76	168 / 79	Neonates & Infants: 45 (59%) Childhood: 22 (29%) NR: 9 (12%) Range: Birth 8 yr (med. 1)	Neonates & Infants: 43 (57%) Childhood: 22 (29%) Adulthood: 11 (14%) Range: Birth 25 yr (med. 1)	Focal: (52) Generalized: Myoclonic (10), Spasms (8), GTC (6)	Dravet syndrome (23) DEE (15) Focal epilepsy (14) DEE (9)	Yes: 73 No: 1 NR: 2	Yes: 46 No: 17 NR: 13	Valproate (40) Phenobarbital (21) Lamotrigine (19) Levetiracetam (18) Clonazepam (16) Clobazam (15)	Normal: 21 Abnormal: 40 NR: 15	Normal: 39 Abnormal: 20 NR: 17	Severe DD/ID (58) Autism/ASD (11) Neuroregression (10) Microcephaly (9) Hypotonia (7)
Sudan	13	56 / 19	Neonates & Infants: 4 (31%) Childhood: 7 (54%) Adulthood: 1 (8%) NR: 1 (8%) Range: Birth 36 yr (med. 2)	Childhood: 12 (92%) Adulthood: 1 (8%) Range: 2–32 yr (med. 6)	Generalized: GTC (3), Tonic (2), Spasms (1) Focal: (2) Mixed/other: (6)	Generalized epilepsy (7) West syndrome (2) Focal epilepsy (2) Unspecified (2)	Yes: 13 No: 0 NR: 0	Yes: 3 No: 1 NR: 9	Valproate (3) Levetiracetam (2) Phenobarbital (2) Unspecified (10)	Normal: 0 Abnormal: 4 NR: 9	Normal: 2 Abnormal: 5 NR: 6	Developmental delay (13) Intellectual disability (6) Spasticity (4) Microcephaly (3)
Tunisia	47	182 / 79	Neonates & Infants: 22 (47%) Childhood: 4 (9%) Adulthood: 1 (2%) NR: 20 (43%) Range: Birth 42 yr (med. 1)	Neonates & Infants: 15 (32%) Childhood: 24 (51%) Adulthood: 8 (17%) Range: Birth–33 yr (med. 7)	Generalized: Tonic (7), Clonic (11), GTC (6), Febrile seizures (13), Myoclonic (4), Spasms (4) Focal: (7) Mixed/other: (23)	Pyridoxine-dependent epilepsy (9) DEE (8) GEFS+/Dravet spectrum (7) Generalized epilepsy (8) Focal epilepsy (6)	Yes: 34 No: 10 NR: 3	Yes: 25 No: 1 NR: 21	Pyridoxine (9) Valproic acid (7) Levetiracetam (4) Clonazepam (3) Sodium valproate (3) Unspecified (21)	Normal: 11 Abnormal: 21 NR: 15	Normal: 20 Abnormal: 9 NR: 18	Developmental delay (44) Intellectual disability (13) ADHD (3) Motor retardation (3)
Tanzania	1	​	Neonates & Infants: 1 (100%)	Childhood: 1 (100%)	Focal: (1)	DEE (1)	Yes (1)	Yes (1)	Diazepam/ valproate (1)	Abnormal: 1	NR :1	Developmental delay
CUMULATIVE TOTAL	384	1340 / 518	-	-	-	-	Yes: 367 No: 12 NR: 5	Yes: 139 No: 23 NR: 222	-	Normal: 75 Abnormal: 199 NR: 110	Normal: 133 Abnormal: 150 NR: 101	-

Abbreviations: Neonates & Infants (Birth-2 yr), Childhood (2-17 yr), Adulthood (≥18 yr), ASM, anti-seizure medication; DD, developmental delay; DEE, developmental and epileptic encephalopathy; GEFS+, genetic epilepsy with febrile seizures plus; GTC, generalized tonic–clonic; ID, intellectual disability; ILAE, International League Against Epilepsy; IS, infantile spasms; NR, not reported; PME, progressive myoclonic epilepsy; VPA, valproic acid.

Regarding epilepsy diagnosis, generalized epilepsy was the most common, 30.5% (n = 117/384), followed by focal, 18.5% (n = 71/384), and unspecified in 10.4% (n = 40/384) of the cases. Among epilepsy syndromes, unspecified DEE was the most frequent, 31.8% (n = 122/384), followed by Dravet syndrome, 7.8% (n = 30/384), and Progressive Myoclonic Epilepsy, 3.6% (n = 14/384). Moreover, rare syndromes such as Lafora disease, Rett syndrome, neuronal ceroid lipofuscinosis, tuberous sclerosis complex, GLUT1 deficiency syndrome, and Sjögren–Larsson syndrome were also reported (Supplementary Table S3). The common seizure types reported were spasms, 20.8% (n = 80/384), focal, 16.4% (n = 63/384), and generalized tonic-clonic, 18.2% (n = 70/384). Other types of seizure were also described, including absence, atonic, and myoclonus seizures. However, the seizure nature was not reported in 7.6% (n = 29/384) of families (Supplementary Table S4).

Neurodevelopmental delay was reported in 95.6% (n = 367/384) families, not found in 3.1% (n = 12/384) and not reported in 1.3% (n = 5/384). Epilepsy was drug-resistant in 36.2% (n = 139/384), drug-sensitive in 6.0% (n = 23/384), and data were not reported in 57.8% (n = 222/384). The commonly used antiseizure medications were valproic acid, phenobarbital, levetiracetam, and lamotrigine.

EEG data were reported in at least one patient in 71.4% (n = 274/384) of families and were abnormal in 72.6% (n = 199/274) of those families. Brain imaging, including CT and MRI, was reported in at least one patient per family in 73.7% (n = 283/384) of families with abnormality in 53.0% (n = 150/283).

Genetic findings (See also Supplementary Table S2).

### Gene distribution across countries

The genetic heterogeneity varied from 1.1 to 3.2 families per gene among the 384 families and 165 unique genes. South Africa exhibited the highest absolute genetic diversity with 24 unique genes identified across 76 families (index: 3.2), followed by Egypt with 65 genes in 144 families (2.2), Morocco with 51 genes in 60 families (1.2), and Mali with 29 genes in 31 families (1.1). One hundred thirty-six genes were limited to single countries, representing 82.4% (n = 136/165) of genes with defined country distribution, appearing in multiple unrelated families within specific populations (Figure 2). Twelve genes were shared across three or more countries (7.3%, n = 12/165), while 17 genes were shared across exactly two countries (10.3%, n = 17/165). In Egypt, 47 genes appeared exclusively in unrelated Egyptian families. *SCN1A* was identified across 5 countries (South Africa, Tunisia, Morocco, Egypt, and Nigeria), representing 11.5% (n = 44/384) of the total cohort. In South Africa, *SCN1A* was identified in 38.2% (n = 29/76) of families. Country-specific genes with the highest recurrence included: *ASAH1* (n = 14 unrelated families, Egypt only; 9.7% of Egyptian cohort (n = 14/144), 3.6% of total cohort (n = 14/384)), *ALDH7A1* (n = 9 unrelated families, Tunisia only; 19.1% of Tunisian cohort (n = 9/47), 2.3% of total cohort (n = 9/384)), *SNX14* (n = 8 unrelated families, Egypt only; 2.1% of total cohort (n = 8/384)), *TRAPPC4* (n = 8 unrelated families, Egypt only; 2.1% of total cohort (n = 8/384)), *CSTB* (n = 8 unrelated families, Egypt only; 2.1% of total cohort (n = 8/384)), and *MLC1* (n = 5 unrelated families, Sudan only; 38.5% of Sudanese cohort (n = 5/13), 1.3% of total cohort (n = 5/384)). Additional country-specific genes included: *ACBD6* (n = 4 unrelated Egyptian families; 1.3% of total cohort (n = 4/383)), *PNKP* (n = 3 unrelated Egyptian families; 0.8% of total cohort (n = 3/384)), *ITPA* (n = 3 unrelated Egyptian families; 0.8% of total cohort (n = 3/384)), *PIDD1* (n = 3 unrelated Egyptian families; 0.8% of total cohort (n = 3/384)), *VARS* (n = 3 unrelated Egyptian families; 0.8% of total cohort (n = 3/384)), and *PGAP3* (n = 3 unrelated South African families; 0.8% of total cohort (n = 3/384)). One novel candidate gene, *TOP3B*, was identified through a 254-kb deletion in a Tunisian family with juvenile myoclonic epilepsy (Supplementary Table S2).

**FIGURE 2:**
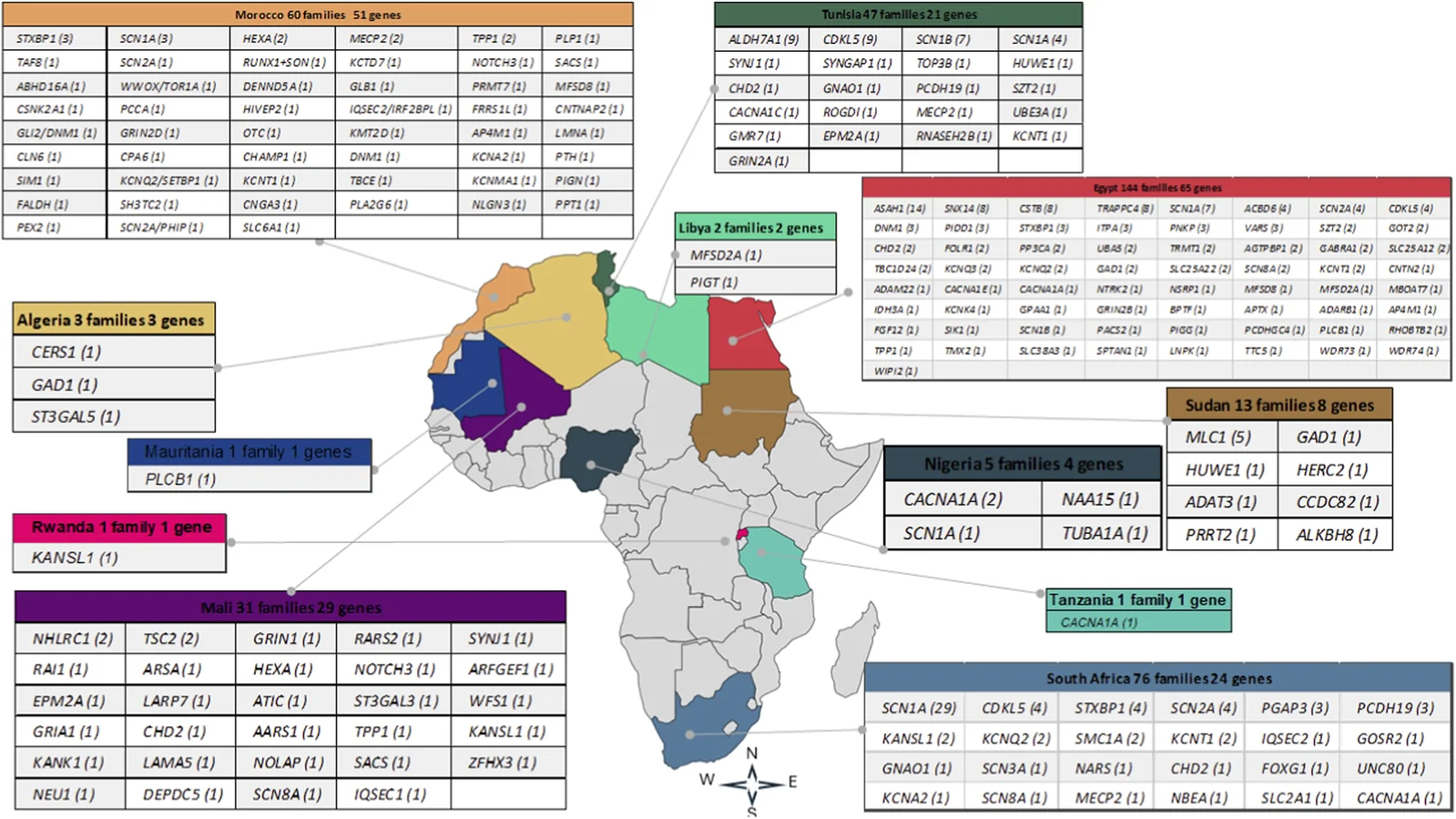
Geographic distribution of genetically characterized epilepsy families and associated genes across Africa. Map of Africa illustrates the distribution of families with a confirmed genetic diagnosis of epilepsy across 12 countries, as identified from the 78 included studies. Each country panel lists the implicated genes alongside the number of families carrying variants in each gene (in parentheses). Countries are color-coded for visual distinction. The number of families and unique genes identified per country are indicated in each panel header.

### Variant types, zygosity, and novelty

A total of 395 variants were identified across all families. Missense variants were the most common, 53.9% (n = 213/395), followed by frameshift, 12.7% (n = 50/398), nonsense, 12,7% (n = 50/395), and splice site variants, 9.9% (n = 39/395). CNVs/structural variants accounted for 7.3% (n = 29/395), while structural rearrangements were identified in 2.3% (n = 9/395) (Figure 3A).

**FIGURE 3:**
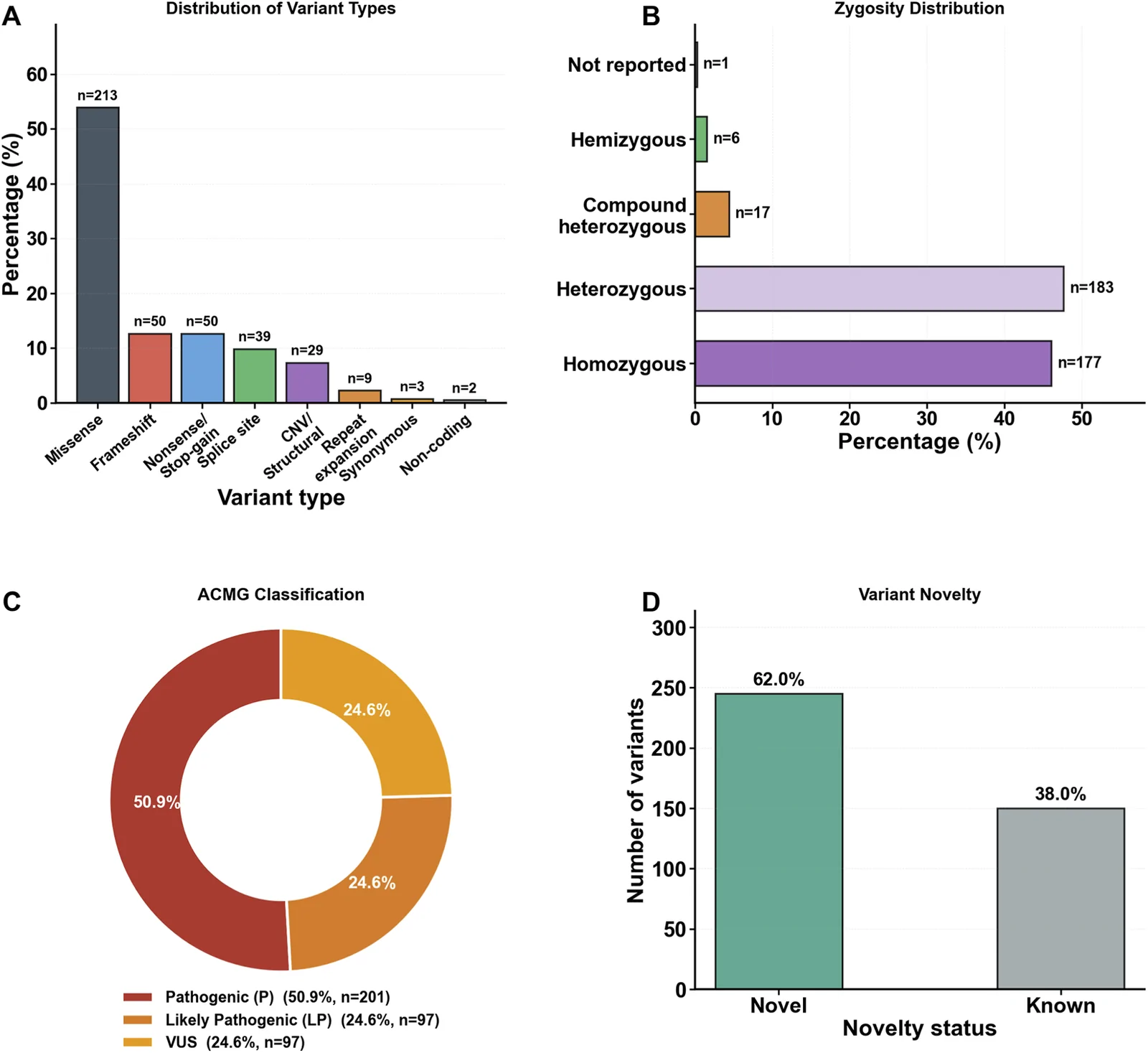
Characterization of identified genetic variants. **(A)** Bar chart showing the distribution of variant types identified across all included studies (total = 395 variants). Missense variants were the most prevalent (n = 213), followed by frameshift (n = 50), nonsense/stop-gain (n = 50), splice site (n = 39), CNV/structural (n = 29), repeat expansion (n = 9), synonymous (n = 3), and non-coding variants (n = 2). **(B)** Horizontal bar chart depicting the zygosity distribution of identified variants. Homozygous (n = 177) and heterozygous (n = 183) variants were the most common, with smaller proportions of compound heterozygous (n = 17), hemizygous (n = 6), and one unreported case. **(C)** Donut chart illustrating ACMG/AMP variant classification. About half of variants were classified as pathogenic (P; 50.9%, n = 201), followed by likely pathogenic (LP; 24.6%, n = 97) and variants of uncertain significance (VUS; 24.6%, n = 97). **(D)** Bar chart showing variant novelty status. Most identified variants were novel (62.0%, n = 245), while 38.0% (n = 150) were previously reported.

Overall, homozygosity was reported in 46.1% of families (n = 177/384) and heterozygosity in 47.7% (n = 183/384), with compound heterozygosity in 4.4% (n = 17/384) and hemizygosity (X-linked) in 1.6% (n = 6/384) (Figure 3B). The highest proportion of homozygous variants was reported in Egypt (60% of cases), where consanguinity was reported in 65.3% (n = 94/144) of families. These variants occurred exclusively in four genes: *ASAH1*, *TRAPPC4*, *CSTB*, and *SNX14*. Similarly, in Sudan, consanguinity was reported in 84.6% (n = 11/13) of families, with five families carrying *MLC1* homozygous variants. In Tunisia, homozygous inheritance was found in *ALDH7A1* (n = 9/9 families, all with reported consanguinity), and heterozygous inherited (autosomal dominant) variants in SCN1B-related febrile seizures (n = 6/7 families). Morocco showed 41.7% (n = 25/60) consanguineous families and 58.3% (n = 35/60) non-consanguineous families. South Africa displayed heterozygous variants in most cases, 93.4% (n = 71/76). Compound heterozygous variants were identified in Egypt (*GPAA1, CDKL5*), Tunisia (*SYNJ1*), Morocco (multiple genes), and Mali (*RARS2, LARP7, AARS1, SACS*) (Supplementary Table S2).

As reported by the individual publications, 50.9% (n = 201/395) of the variants were classified by ACMG criteria as pathogenic, 24.6% (n = 97/395) were likely pathogenic, and 24.6% (n = 97/395) were variants of uncertain significance in established epilepsy genes and were thus reported as candidate variants associated with the patient’s epilepsy (Figure 3C). A total of 293/384 (76.3%) of families were found to have pathogenic or likely pathogenic variants, with 91/384 (23.7%) having VUSs in genes consistent with the seizure phenotype.

The majority of variants were novel (62.0%, n = 245/395), while 38.0% (n = 150/395) were previously reported (Figure 3D). The proportion of novel variants varied by country: Egypt 61.1% (n = 88/144), Mali 80.6% (n = 25/31), Morocco 55.0% (n = 33/60), and Tunisia 72.3% (n = 34/47). Among novel variants, 67.8% (n = 166/245) were classified as pathogenic or likely pathogenic, while 32.2% (n = 79/245) were classified as VUS.

Of the 97 VUS, an inheritance pattern was reported for 93 (95.9%). Of these, 25 (25.8%) were reported as *de novo*, 58 (59.8%) as autosomal recessive (predominantly homozygous), 6 (6.2%) as autosomal dominant, and 4 (4.1%) as X-linked; 4 (4.1%) had no inheritance information reported. All 97 VUS occurred in genes with an established epilepsy gene–disease association. Twelve of the 97 VUS (12.4%) were located in genes with reported precision anti-seizure medication implications, including sodium channelopathy genes (*SCN1A, SCN2A, SCN3A*), potassium channelopathy genes (*KCNQ2, KCNQ3*), and glutamate receptor genes (*GRIN1, GRIN2D*). Country-specific founder variants contributed to known variant enrichment in specific populations. This includes Tunisia with the highest proportion of known variants in *ALDH7A1* and *SCN1B*. In addition, Egypt had a recurrent known variant in *CSTB* (expansion of the dodecamer (CCCCGCCCCGCG) repeat, in 8 families).

Only two identical pathogenic variants were shared across countries: *SCN1A* (c.4547C > A: p. Ser1516Ter) and *KCNT1* (c.862G > A: p. Gly288Ser), both identified in South Africa and Egypt. Two founder variants were identified in Tunisia: *SCN1B*: c.374G > A in 7 families and *ALDH7A1*: c.1364T > C in 9 families. Four founder variants were identified in Egypt: *TRAPPC4*: c.427A > G in 8 families; *CSTB* dodecamer expansion in 8 families; *SNX14*: c.-24C > T (rs786205229) in 3 families; and *ASAH1*: c.155T > C in 8 families (Supplementary Table S2).

### Inheritance pattern

Autosomal recessive inheritance was predominant, 49.5% (n = 190/384), followed by *de novo* variants, 32.0% (n = 123/384). Inheritance was not reported or unknown in 8.9% (n = 34/384). Autosomal dominant was identified in 4.7% (n = 18/384), X-linked recessive in 2.9% (n = 11/384), and X-linked *de novo* in 2.1% (n = 8/384) (Supplementary Figure S1D).

### Diagnostic methods and yield

Among 78 studies identified, only the cohort studies (9.0%, n = 7/78) reported diagnostic yield, which ranged from 17.5% to 43.9% (Figures 4A,B). Whole exome sequencing (WES) was the predominant diagnostic method, 71.8% (n = 56/78), followed by Sanger sequencing, 10.3% (n = 9/78), gene panels, 6.4% (n = 5/78), cytogenetic methods, 5.1% (n = 4/78), and long-read/whole genome sequencing, 3.8% (n = 3/78). The use of WES increased substantially from the year 2019 onwards, with publication output (n = 12) peaking in 2024 (Figure 4C).

**FIGURE 4:**
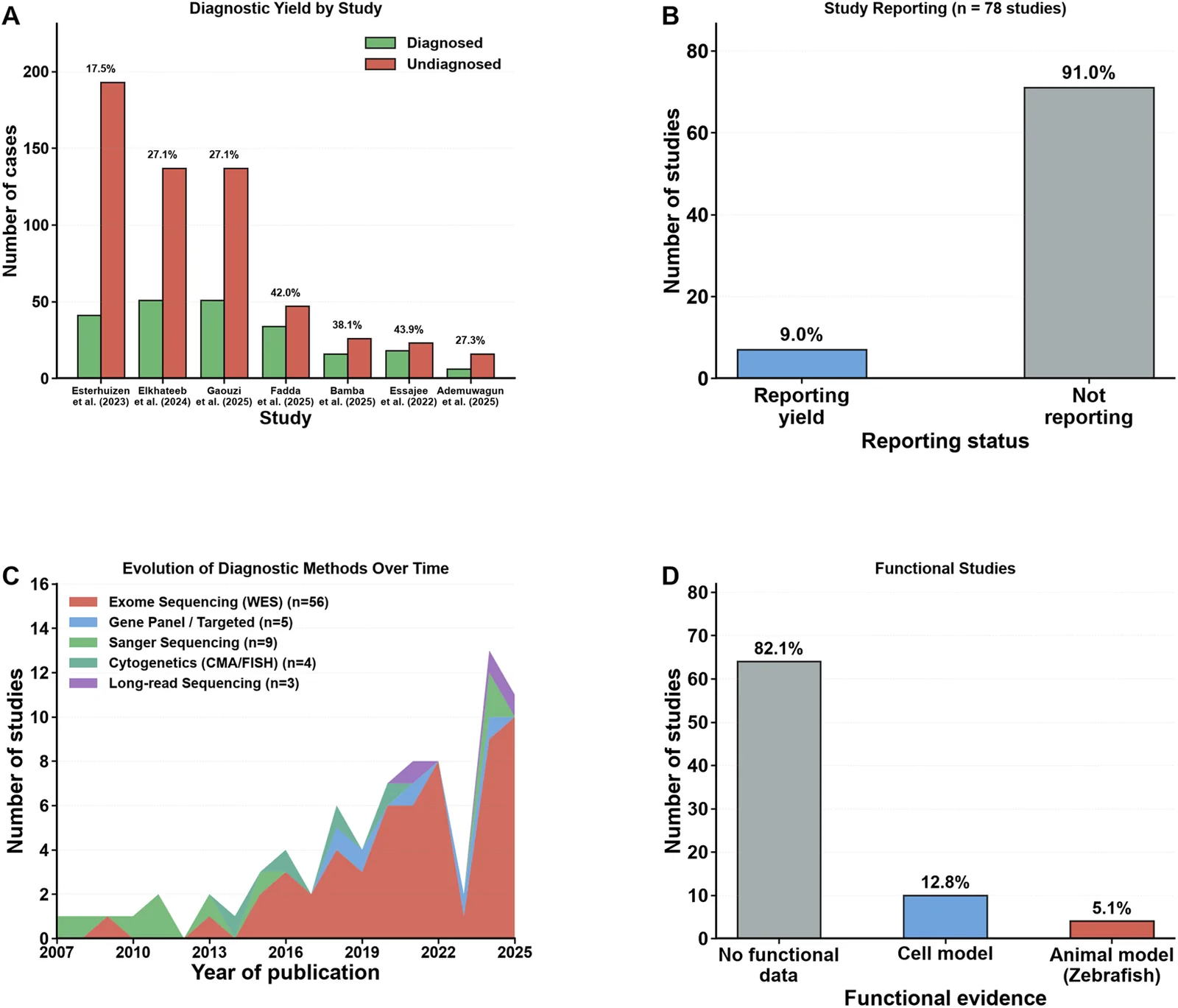
Diagnostic yield, reporting practices, sequencing methods, and functional evidence. **(A)** Grouped bar chart showing the diagnostic yield (percentage of diagnosed vs. undiagnosed cases) for seven studies that explicitly reported this outcome. Diagnostic yields ranged from 17.5% (Esterhuizen et al., 2023) to 43.9% (Essajee et al., 2022). **(B)** Bar chart showing the proportion of included studies (n = 78) that reported diagnostic yield. Only 9% of studies reported yield, while 91% did not. **(C)** Stacked area chart depicting the evolution of genomic diagnostic methods used across studies from 2007 to 2025. Exome sequencing (WES) was the dominant method (n = 56), with increasing adoption of long-read sequencing in recent years alongside Sanger sequencing, gene panels, and cytogenetics (CMA/FISH). **(D)** Bar chart illustrates the availability of functional evidence in included studies. The majority (82.1%) reported no functional data, while 12.8% included cell model validation and 5.1% used animal models (zebrafish).

### Functional studies

Functional validation of variants was performed in 17.9% of studies (n = 14/78), including cell models, 12.8% (n = 10/78) and animal models, 5.1% (n = 4/78) (Figure 4D). Cell-based studies primarily used patient-derived fibroblasts, lymphoblasts, leukocytes, neural progenitor cells, HEK293-derived expression systems, and transfected cell lines to investigate variant pathogenicity and underlying molecular mechanisms. Animal studies employed zebrafish and mouse models to evaluate neurodevelopmental abnormalities, seizure susceptibility, and therapeutic response. Most functional studies were performed in laboratories outside Africa through international collaborations except for one study with cell-based assays of *PIGT* that was conducted in Tunisia in collaboration with a laboratory in Japan.

### Gene functional category

After excluding CNV, we further categorized the total number of variants in the remaining 362 families with identified pathogenic variants based on gene functional pathways. Ion channel genes were the most frequent functional category, 27.3% (n = 99/362), followed by synaptic function genes, 15.2% (n = 55/362), lysosomal storage genes, 9.4% (n = 34/362), metabolism genes, 7.7% (n = 28/362), membrane trafficking genes, 6.6% (n = 24/362), chromatin/transcription genes, 4.4% (n = 16/362), cell signaling genes, 4.1% (n = 15/362), and DNA repair genes, 1.7% (n = 6/362) (Supplementary Figure S1E). Eighty-five families had genes that remained unclassified into these functional categories, 23.5% (n = 85/362).

### Phenotype-genotype correlations

The analysis across 384 families revealed high phenotypic consistency for specific gene-syndrome pairs regardless of geographic origin of the patients. *SCN1A* variants were consistently associated with Dravet syndrome across 5 countries, 79.5% (n = 35/44). Dravet syndrome was characterized by early-onset focal seizures, severe developmental delay in all patients (100%, n = 44/44), and drug resistance in 61.4% (n = 27/44). In addition, there was a phenotype-genotype correlation between *ALDH7A1* and neonatal-onset seizures responsive to pyridoxine supplementation in Tunisia in 100% (n = 9/9) of cases. *CDKL5* variants consistently caused early infantile epileptic encephalopathy with hypsarrhythmia in all cases (100%, n = 17/17) across three countries (South Africa (n = 4), Tunisia (n = 9), Egypt (n = 4)). Furthermore, country-specific phenotypic patterns showed that in Egypt, DEE with epileptic spasms was the predominant presentation with 47.2% (n = 68/144), caused by genes including *ASAH1* (20.6%, n = 14/68), *TRAPPC4* (11.8%, n = 8/68), *VARS* (4.4%, n = 3/68), and *PIDD1* (4.4%, n = 3/68). Tunisia exhibited unique enrichment for treatable pyridoxine-dependent epilepsy in 19.1% (n = 9/47), exclusively caused by biallelic *ALDH7A1* variants (9 families, all autosomal recessive). South Africa showed a high prevalence of Dravet syndrome, 30.3% (n = 23/76), and drug resistance, 60.9% (n = 14/23), predominantly attributed to *SCN1A* variants, 38.2% (n = 29/76) (Supplementary Table S1).

## Discussion

### Study characteristics and epilepsy research dynamics

This study provides a comprehensive analysis of published literature on genetic epilepsies across Africa, encompassing 78 studies from 12 nations reporting 519 individuals with genetic epilepsy from 384 families. Remarkably, 90% of the reported families come from just five of the 54 countries in the continent, Egypt, South Africa, Morocco, Tunisia, and Mali, and more than two-third of African nations have not had a single publication in epilepsy genetics. Although the most common age of onset was in neonates and infants, consistent with observations from international cohorts of genetic epilepsy, molecular diagnosis was frequently delayed until childhood or adulthood in several countries, including Tunisia, Sudan, Libya, Egypt, and especially Nigeria (Ademuwagun et al., 2025; Ben et al., 2018; Ben Said et al., 2024; Ferlazzo et al., 2016; Thakran et al., 2020; Desprairies et al., 2020). This is likely due to the lack of widespread availability of molecular diagnosis capabilities (Elkhateeb et al., 2024). This highlights a substantial research gap in understanding the genetic architecture of epilepsy across the continent, despite Africa’s high burden of epilepsy (Bamba et al., 2025; Bamba et al., 2024; Cisse et al., 2024; Girgis et al., 2024; Neuray et al., 2020; de Sainte Agathe et al., 2025; van der Knoop et al., 2022; Abou El Ella et al., 2018; Heide et al., 2022; Wong et al., 2022; Adedokun et al., 2016).

Several initiatives over the past decade have helped African scientists to study hereditary diseases, including the H3Africa initiative, which has provided critical genomic infrastructure, funding, and collaborative networks (Landouré et al., 2025). It is notable that from 2015 to present, publications have generally increased, with an acceleration after 2020 and a peak in 2024, despite potential disruptions during the *COVID-19* pandemic.

### Phenotypic spectrum of epilepsy in Africa

The most common epilepsy seizure type identified in this review was generalized tonic-clonic seizures, consistent with previous studies (Perucca et al., 2020). Among specific genetic syndromes, DEE and Dravet syndrome were the most frequently reported (Adedokun et al., 2016; Charfi et al., 2024; Esterhuizen et al., 2018; Chatron et al., 2020; Jdila et al., 2021; Sahli et al., 2019; El Mouhi et al., 2024; Fendri-Kriaa et al., 2011; Friedman et al., 2019; Essajee et al., 2022). This study also found a significant proportion of drug-resistant epilepsy. This is not surprising, owing to the higher proportion of developmental and epileptic encephalopathies, in which pharmaco-resistance is a core feature (Remy and Beck, 2006). Furthermore, the genetic basis of pharmaco-resistance in epilepsy is well documented from recent studies (Remy and Beck, 2006). Altogether, this suggests that there is an urgent need to fully capture the underlying genetic basis of epilepsy for targeted therapeutics. Bridging this treatment gap requires not only genetic diagnosis but also healthcare system strengthening to deliver precision therapies equitably.

### Genetic architecture of epilepsy in Africa

The genetic spectrum of epilepsy has been reported to be highly diverse in other populations and we found 165 unique causative genes across the 293 families with likely pathogenic or pathogenic variants (Stevelink et al., 2023; Exome sequencing of 20, 2024). Causative variants mostly involved genes encoding for ion channels and synaptic function proteins found in 27.1% and 15.2%, respectively. Similar findings were previously reported with ion channel genes being the common causes of genetic epilepsy, representing about 25% (Oyrer et al., 2018). In fact, *SCN1A* is a voltage-gated sodium channel, which encodes the NaV1.1 subunit expressed predominantly in inhibitory GABAergic neurons and is enriched at the axon initial segment. It plays a key role in the initiation and propagation of action potentials in these cells (Yu et al., 2006; Ogiwara et al., 2007; Duflocq et al., 2008). The causal relationship between *SCN1A* and Dravet syndrome, seen in this review, has been well known (Oyrer et al., 2018; Yu et al., 2006).

Other rare genes associated with severe phenotypes like progressive myoclonic epilepsy include Laforin *(EPM2A*) and malin (*NHLRC1*). These genes operate in the same pathway controlling glycogen phosphorylation and degradation (Trao et al., 2009). The identification of PME genes across North African and West African cohorts suggests population-specific patterns which differ from the Finnish and Mediterranean populations (Cisse et al., 2024; Trao et al., 2009; Khiari et al., 2011; Hosny et al., 2021; Anderson et al., 2017). *ACBD6* and *SNX14* were identified with recurrent variants only in Egyptian consanguineous families (Kaiyrzhanov et al., 2024; Akizu et al., 2015). Recent genetic studies have reported novel candidate genes in African families, critically contributing to expanding the genetic spectrum of the disease. Notably, TTTTA repeat expansions and TTTCA repeat insertions in intron 4 of the *RAI1*, causing benign adult familial myoclonic epilepsy (BAFME) type 8 was reported in a Malian family (Yeetong et al., 2024). The study further suggested that haploinsufficiency, previously reported as a common mechanism of BAFMEs, is unlikely to be the main pathomechanism (Yeetong et al., 2024; Landouré et al., 2025). In addition, a homozygous missense variant in *AP2A2* was identified in a consanguineous Malian family hereditary spastic paraplegia accompanied by intellectual disability and seizures. *AP2A2* knockout in *Xenopus tropicalis* model that recapitulated the seizure phenotype observed in patients (Diarra et al., 2024). Another, novel candidate gene *TOP3B* carrying a single 254-kb deletion associated with PME was reported in Tunisian family (Daghsni et al., 2018). However, despite strong segregation and RNA analysis data, there was no functional mechanistic evidence reported, highlighting the challenge of validating a novel candidate gene validation in resource-limited settings. Considering the discovery of previously unreported genes causing epilepsy in such small cohorts, there is the potential to uncover other novel genes in larger cohorts.

Moreover, most reported variants in genes previously associated with epilepsy were novel, suggesting that these might be Africa-specific variants and underscoring the scarcity of African genetic data on the public genomic data repository (Elkhateeb et al., 2024; Girgis et al., 2024; Abou El Ella et al., 2018; Trao et al., 2009; Khiari et al., 2011; Abaied et al., 2010; Maiga et al., 2025; Tlili et al., 2013; T et al., 2013; Ouskri et al., 2025; Ramsay, 2022). Importantly, founder variants were also described in African families. This could have immediate clinical implications, including population-based heterozygous carrier screening in their respective general populations. It will also allow appropriate genetic counseling, and for amenable conditions, intervention could be initiated as early as possible before irreversible neurological damage occurs.

The reported variants were mostly missense, corroborating with previous literature. Another important point is that the consanguinity rate varied across African countries, tightly related to the distribution of the specific types of variants. For instance, homozygous variants were found predominantly in countries with the highest rates of consanguinity practices, including Egypt (103/139 variants, 74%), Sudan (9/10, 90%), Mali (14/31, 45%) and Morocco (21/60, 35%) (Abou El Ella et al., 2018; Heide et al., 2022; Wong et al., 2022; Adedokun et al., 2016; Chatron et al., 2020; Friedman et al., 2019; Hosny et al., 2021; Kaiyrzhanov et al., 2024; Akizu et al., 2015; Mahmoud et al., 2020; Ghosh et al., 2021; Ghosh et al., 2020; Zaki et al., 2021; van Karnebeek et al., 2019; Calame et al., 2021; Musante et al., 2022; Johansen et al., 2016; Makrythanasis et al., 2016; Rosti et al., 2016; Maroofian et al., 2021; Breuss et al., 2018; Nguyen et al., 2017; Marafi et al., 2022; Efthymiou et al., 2025; Stogmann et al., 2013; Elhossini et al., 2024; Yahia et al., 2022; Koko et al., 2024; Fadda et al., 2025; Gaouzi et al., 2025; Bouzroud et al., 2022). Despite the low number of families, Algeria, Libya, and Mauritania displayed high homozygous variant frequencies with 3/3 (100%), 2/2 (100%), and 1/1 (100%), respectively (Thakran et al., 2020; de Sainte Agathe et al., 2025; Abou El Ella et al., 2018; Chatron et al., 2020; Guemez-Gamboa et al., 2015; Ben Ayed et al., 2023). On the other hand, heterozygous variants, predominantly *de novo,* were mostly reported in South Africa (63/68, 93%), Nigeria (5/5, 100%), Rwanda (1/1, 100%), and Tanzania (1/1. 100) (Ademuwagun et al., 2025; Uwineza et al., 2014; Esterhuizen et al., 2023; Chipongo et al., 2024). However, Morocco and Mali showed a more diverse zygosity profile, with 35% (21/60) homozygous, 52% (31/60) heterozygous, 7% (4/60) compound heterozygous, and 5% (3/60) hemizygous variants, and 45% (14/31) homozygous, 35% (11/31) heterozygous, 13% (4/31) compound heterozygous, and 3% (1/31) hemizygous variants, respectively. This indicates a greater genetic heterogeneity in these populations than the predominantly homozygous cohorts. In addition, it reflects the fundamental difference in population structure and cultures in countries like South Africa, compared to others where consanguinity practices are more prevalent (Anwar et al., 2014; Bezuidenhout et al., 2020; Mezzi et al., 2024; Chentouf et al., 2015). Diagnosis methods have also evolved gradually in African context, with a sharp shift from target gene sequencing approach to the next-generation sequencing, including exome or genome sequencing (Landoure et al., 2014; Essid et al., 2025; Ashaat et al., 2024; Dafir et al., 2020; Jouali et al., 2025; Bouhouche et al., 2018; Ratbi et al., 2015). However, these tools are not widely available to African researchers, despite the fact that they are recognized as state of art techniques in genetic studies (Symonds and McTague, 2020).

### Strengths and limitations

This study has several strengths. It provides the most comprehensive clinical and genetic spectrum of epilepsy reported so far in Africa. We analyzed the phenotypic and genetic architectures of epilepsy in Africa, including diverse populations. This will provide the genetic spectrum of epilepsy in Africa and could help to design future larger multicentric studies and inform therapeutics perspectives.

However, despite these promising perspectives in this area, there are also some weaknesses to acknowledge. First, the heterogeneous nature of the studies across countries may lead to inconsistency in the phenotype descriptions since different standards have been used. For instance, studies have used different classifications of seizure, since ILAE guidelines have also evolved. In addition, seizure phenotype was not reported for 7.6% of the studies. Together, these factors add some uncertainty to the analysis of phenotype-genotype correlation. Second, the hospital-based nature of the cohorts could potentially limit their generalizability. Third, these data might not reflect the true clinical and genetic architectures of epilepsy in Africa since reports were identified in only 12 out of 54 African countries, and data published in local non-indexed journals might have not been captured. Lastly, the language restriction to English and French might exclude articles published in other languages and can potentially cause bias. However, based on the number of countries covered by these two languages (> 90%), it is unlikely that a significant number of articles are missed.

### Future directions

African populations are critically underrepresented in genomic databases, which impacts variant classification and clinical interpretation (Quansah and McGregor, 2018; Landouré et al., 2025). In addition, there are clear inequities both between African countries and between African countries and developed countries. This is not specific to epilepsy research but more broadly reflects genetic research capabilities (Alimohamed et al., 2026).

While important progress has been achieved in epilepsy research in Africa, a significant effort is still needed to accurately delineate the genetic underpinnings. This involves strengthening research infrastructures, training specialized healthcare personnel, and integration of genetic services into existing healthcare systems across the continent (Landouré et al., 2025). To sustain the current effort, leaders and stakeholders, especially in sub-Saharan Africa, should devote resources towards training young neuroscientists, geneticists, and neurologists and leverage regional centers of excellence to create databases specific to Africa. Several national societies are stepping up to build nationwide genome projects to facilitate this process (Yalcouyé et al., 2026). Despite challenges outlined above that hinder genomics research in Africa, the continent’s unprecedented diversity provides ample opportunity for future precision medicine discoveries (Landouré et al., 2025; Alimohamed et al., 2026). On a broader scale, a recent review suggested that a coordinated agenda is essential to build research capacity, generate data, foster equitable collaborations and translate discoveries into meaningful clinical care for hereditary neurological diseases. Strong commitment to the outlined strategies is essential to advance research on not only epilepsy but all hereditary disorders in Africa (Landouré et al., 2025).

## Conclusion

This study describes the current profile of genetic epilepsy in Africa, highlighting notable clinical and genetic heterogeneity. Most variants reported are novel and/or population specific. While important progress has been made, substantial gaps remain compared to developed countries in terms of access to genomic technologies, particularly in sub-Saharan Africa, where most countries lack molecular diagnostic capabilities. Notably, most reported studies in this review are from North Africa and these data may therefore not reflect the full landscape of genetic epilepsy of the African continent. There is urgent need to address these gaps by conducting multicenter and large-scale studies involving different regions of Africa. The involvement of African researchers in international epilepsy efforts, such as through ILAE or Cure Epilepsy, also carries the potential to boost the inclusion of African populations in global epilepsy genetic research. This study could serve as a strong foundation for such efforts.
