# Supplementary material for: Epilepsy genetics in Africa: a systematic review
Source: Front Genet. 2026 Oct 28;17:1964157. doi: 10.3389/fgene.2026.1964157 (PMC13752692; doi:10.3389/fgene.2026.1964157)
Supplement: Supplementary file 1 [file Table3.docx]

**Table S3**: Seizure type reported in African patients

| **Seizure type** | **n** | **proportion** | **percent** |
| --- | --- | --- | --- |
| Epileptic spasms | 80 | 0.208333 | 20.8 |
| Focal | 64 | 0.166667 | 16.7 |
| Generalized tonic-clonic | 72 | 0.187500 | 18.8 |
| Myoclonic | 29 | 0.075521 | 7.6 |
| Myoclonic, Generalized tonic-clonic | 17 | 0.044271 | 4.4 |
| Unspecified | 32 | 0.083333 | 8.3 |
| Tonic | 14 | 0.036458 | 3.6 |
| Clonic | 10 | 0.026042 | 2.6 |
| Absence | 9 | 0.023438 | 2.3 |
| Febrile seizures | 9 | 0.023438 | 2.3 |
| Myoclonic, Atonic | 6 | 0.015625 | 1.6 |
| Myoclonic, Tonic | 6 | 0.015625 | 1.6 |
| Tonic, Focal | 5 | 0.013021 | 1.3 |
| Convulsive seizures | 3 | 0.007813 | 0.8 |
| Generalized tonic-clonic, Focal | 4 | 0.010417 | 1.0 |
| Absence, Myoclonic | 2 | 0.005208 | 0.5 |
| Epileptic spasms, Tonic | 2 | 0.005208 | 0.5 |
| Febrile seizures, Myoclonic, Atonic | 2 | 0.005208 | 0.5 |
| Myoclonic, Tonic, Focal | 2 | 0.005208 | 0.5 |
| Status epilepticus | 2 | 0.005208 | 0.5 |
| Absence, Atonic, Tonic | 1 | 0.002604 | 0.3 |
| Absence, Focal | 1 | 0.002604 | 0.3 |
| Atonic | 1 | 0.002604 | 0.3 |
| Clonus of lower limbs then upper | 1 | 0.002604 | 0.3 |
| DD, autism | 1 | 0.002604 | 0.3 |
| Dravet | 1 | 0.002604 | 0.3 |
| Epileptic spasms, Myoclonic, Tonic | 1 | 0.002604 | 0.3 |
| Febrile seizures, Clonic, Focal | 1 | 0.002604 | 0.3 |
| Febrile seizures, Generalized tonic-clonic | 1 | 0.002604 | 0.3 |
| Febrile seizures, Tonic | 1 | 0.002604 | 0.3 |
| GEFS+ | 1 | 0.002604 | 0.3 |
| Myoclonic, Focal | 1 | 0.002604 | 0.3 |
| Myoclonic, Tonic, Clonic | 1 | 0.002604 | 0.3 |
| Tonic, Clonic, Focal | 1 | 0.002604 | 0.3 |
| Total | **384** |  | 100 |
