# Supplementary material for: Epilepsy genetics in Africa: a systematic review
Source: Front Genet. 2026 Oct 28;17:1964157. doi: 10.3389/fgene.2026.1964157 (PMC13752692; doi:10.3389/fgene.2026.1964157)
Supplement: Supplementary file 2 [file Table4.docx]

**Table S4:** Genetic epilepsy type described in Africa

| **Epilepsy syndrome** | **n** | **proportion** | **percent** |
| --- | --- | --- | --- |
| Generalized epilepsy | 117 | 0.315364 | 31.5 |
| Focal epilepsy | 70 | 0.188679 | 18.9 |
| Dravet syndrome | 44 | 0.118598 | 11.9 |
| Unspecified | 40 | 0.107817 | 10.8 |
| DEE | 24 | 0.064690 | 6.5 |
| STXBP1-DEE | 10 | 0.026954 | 2.7 |
| Pyridoxine-dependent epilepsy | 9 | 0.024259 | 2.4 |
| GEFS+/Dravet spectrum | 8 | 0.021563 | 2.2 |
| CDKL5-DEE | 7 | 0.018868 | 1.9 |
| Progressive Myoclonic Epilepsy (PME) | 7 | 0.018868 | 1.9 |
| Developmental and Epileptic Encephalopathy (DEE) | 5 | 0.013477 | 1.3 |
| West syndrome | 5 | 0.013477 | 1.3 |
| KCNQ2-DEE | 4 | 0.010782 | 1.1 |
| Lafora disease | 4 | 0.010782 | 1.1 |
| PCDH19 Epilepsy | 4 | 0.010782 | 1.1 |
| Rett syndrome | 4 | 0.010782 | 1.1 |
| Neuronal Ceroid Lipofuscinosis | 3 | 0.008086 | 0.8 |
| Tuberous Sclerosis Complex | 2 | 0.005391 | 0.5 |
| Febrile seizures | 1 | 0.002695 | 0.3 |
| Focal epilepsy with speech disorder | 1 | 0.002695 | 0.3 |
| GLUT1 deficiency syndrome | 1 | 0.002695 | 0.3 |
| Sjögren–Larsson syndrome | 1 | 0.002695 | 0.3 |
